# Comparison of hypoxemia, intubation procedure, and complications for non-invasive ventilation against high-flow nasal cannula oxygen therapy for patients with acute hypoxemic respiratory failure: a non-randomized retrospective analysis for effectiveness and safety (NIVaHIC-aHRF)

**DOI:** 10.1186/s12873-021-00402-w

**Published:** 2021-01-14

**Authors:** Chao Zhang, Min Ou

**Affiliations:** Department of the Sixth Health Care, The Sixth Department of Health Care, The Second Medical Center & National Clinical Research Center for Geriatric Diseases, Chinese PLA General Hospital, Beijing, 100048 China

**Keywords:** High-flow oxygen therapy, Hypoxia, Laryngoscopy, Intubation, Non-invasive ventilation, Preoxygenation

## Abstract

**Background:**

Optimization of preoxygenation procedure can help to secure the method of intubation by reducing the risks of severe hypoxemia and other problems. There is confusion for efficacy of non-invasive ventilation compared to high-flow oxygen therapy regarding occurrence of severe hypoxemia during the intubation procedure. The purpose of the study was to compare the difference between noninvasive ventilation and high flow oxygen therapy to prevent desaturation during laryngoscopy.

**Methods:**

Patients underwent high-flow nasal cannula oxygen therapy (HCO cohort, *n* = 161) or non-invasive ventilation procedure (NIV cohort, *n* = 154) for oxygenation and ventilation due to acute hypoxemic respiratory failure in the intensive care unit. Data before preoxygenation, preoxygenation, intubation, laryngoscopy, and complications of patients due to tracheal intubation were retrospectively collected and analyzed.

**Results:**

There was no difference between both cohorts for the demographical and clinical conditions of the patients before preoxygenation (*p* > 0.05 for all parameters), numbers of patients with severe hypoxia during the intubation procedure (35 vs. 45, *p* = 0.303), the time duration of laryngoscopy (*p* = 0.847), number of laryngoscopies attempts (*p* = 0.804), and immediate and late complications during the intubation procedure. The values of pulse oximetry were reported higher for patients of NIV cohort than those of HCO cohort during preoxygenation. Fewer numbers of patients were reported with severe hypoxia among patients of the NIV cohort than those of the HCO cohort (24 vs., 40, *p* = 0.042) who have moderate-to-severe hypoxemia (partial pressure of arterial oxygen to fraction of inspired oxygen ratio ≤ 200 mmHg) before preoxygenation. The most common complications were hypertension, pulmonary aspiration, and increased 30-day mortality.

**Conclusions:**

When compared, there was no difference between non-invasive ventilation technique and high-flow oxygen therapy to minimize severe hypoxia prior to laryngoscopy and endotracheal intubation in patients with acute respiratory failure.

## Background

Tracheal intubation is commonly used in intensive care units [1]. The tracheal intubation procedure is safe and complications are rare in clinical practice but are fatal. It has a complications like severe hypoxemia, cardiac or neurological ischemia, and cardiovascular collapse especially in the intensive care units [2]. Among patients admitted in the intensive care units, the severe hypoxemia may occur in 20–25% of patients [3] and cardiac arrest may occur in 2–3% of patients [4] and most of them are intubated for acute respiratory failure purpose. Optimization of the preoxygenation procedure can help to secure the method of intubation by reducing the risks of severe hypoxemia and other problems [2].

Oxygenation devices used mostly in the intensive care units are non-invasive ventilation (NIV) and high-flow nasal cannula oxygen therapy (high-flow oxygen; HCO) and that can provide a higher fraction of inspired oxygen than standard oxygen through the Bag Valve Mask [5, 6]. HCO is able to deliver constant high gas flow through nasal prongs up to 70 L/ min, resulting in a high (> 0.9) fraction of inspired oxygen, which is similar to that of the reservoir Bag Valve Mask [7]. During the apneic phase of intubation after anesthetic induction, HCO can maintain oxygenation and avoid hypoxemia. However, NIV is removed during the apneic phase of intubation after anesthetic induction [2]. A prospective study [8] and a randomized trial [9] are reported that during intubation, compared with the reservoir Bag Valve Mask for oxygenation and ventilation, HCO is able to decrease the incidences of severe hypoxemia during intubation procedure. While the randomized controlled trials [10–12] are not provided satisfactory results for HCO compared to the Bag Valve Mask for preoxygenation. However, a randomized trial on acute hypoxemic respiratory failure patients [2] is reported that NIV and HCO both are equally effective. Therefore, there is confusion for efficacy of NIV compared to HCO regarding occurrence of severe hypoxemia during the intubation procedure.

The objective of the non-randomized retrospective study was to compare NIV against HCO regarding occurrences of hypoxemia, intubation procedure, and laryngoscopy procedure in patients undergoing tracheal intubation due to acute hypoxemic respiratory failure admitted into the intensive care units.

## Methods

### Study population

Patients (> 18 years age) undergoing tracheal intubation (under NIV or HCO) due to acute hypoxemic respiratory failure (signs of respiratory distress) into the intensive care units were included in the analysis. Patients less than 18 years in age, patients who were admitted to the operating room and underwent tracheal intubation, and patients who had a Glasgow coma score < 8 were excluded from the analysis.

### Non-invasive ventilation procedure

Here preoxygenation was performed through a face mask connected to the intensive care unit ventilator by Bi-level Positive Airway Pressure machine (GE Healthcare, Chicago, IL, USA). The pressure-support of ventilation was adjusted to get a 6–8 mL/ kg expired tidal volume, 10 cm H_2_O positive end-expiratory pressure, and 1.0 fraction of inspired oxygen. NIV procedure was continued to provide oxygenation and ventilation during preoxygenation and between induction and laryngoscopy. NIV procedure did not continue to provide oxygenation and ventilation during laryngoscopy.

### High-flow nasal cannula oxygen therapy

Preoxygenation was performed by oxygen continuously through nasal prongs, with a 60 L/ min gas flow by a heated humidifier (Apex Medical Corporation, Taipei, Taiwan) and 1.0 fraction of inspired oxygen. The emergency physician(s) was performed a jaw thrust to maintain an upper airway of the patient and high-flow oxygen therapy was continued during laryngoscopy until the endotracheal tube was inserted into the trachea. This therapy was continued to provide oxygenation and little ventilation during preoxygenation, between induction and laryngoscopy, and during laryngoscopy.

### Preoxygenation

Preoxygenation was done in a semi-recumbent position of patients at 30 °C for 3–5 min. Intubation care bundle management was included the pre-intubation presence of two operators, systematic fluid loading (500 mL normal saline (Baxter pharmaceuticals, Deerfield, Illinois, USA)) using 0.2–0.3 mg/ kg etomidate (Etomidate-Lipuro, B. Braun Melsungen AG, Melsungen, Germany) or 1.5–3 mg/ kg ketamine (Ketalar®, Par Pharmaceutical Chestnut Ridge, NY, USA), combined with 0.6–1 mg/ kg rocuronium (Fresenius-Kabi Inc., Lake Zurich, IL, USA) or 1 mg/ kg succinylcholine (Anectine®, Sandoz, Sanofi-Aventis, Princeton, NJ, USA) [3]. If intubation was not successful then video laryngoscopy was adopted. If video laryngoscopy was not successful then surgical tracheostomy (using Tracheostomy tube, F. Hoffmann-La Roche AG, Basel, Switzerland) was adopted. After endotracheal intubation, patients were mechanically ventilated (CARAT II PRO, Hoffrichter GmbH, Mettenheimerstraße, Schwerin, Germany) at 6–8 mL/ kg tidal volume, 25–30 breaths/ min respiratory rate, 5 cm H_2_O positive end-expiratory pressure, and 1.0 fraction of inspired oxygen to maintain 90% or above pulse oximetry (Masimo, Irvine, CA, USA). The partial pressure of arterial oxygen to fraction of inspired oxygen ratio was calculated as per Eq. (1) [2].

1 $$ \mathrm{Fraction}\ \mathrm{of}\ \mathrm{inspired}\ \mathrm{oxygen}=0.21+\mathrm{oxygen}\ \mathrm{flow}\ \mathrm{rate}\times 0.03. $$

### Cohorts

Patients who had recent laryngeal, esophageal, or/ and substantial facial fractures underwent HCO therapy (HCO cohort) otherwise all patients underwent NIV technique (NIV cohort) for acute hypoxemic respiratory failure in the intensive care unit.

### Simplified acute physiology score II

It was calculated from 17 variants before preoxygenation from the information about medical history. Scores are ranged from 0 to 163, with higher the scores a more severe diseased condition [13].

### Modified Cormack-Lehane grade

Modified Cormack-Lehane grade was evaluated in the range from I to IV. If the vocal cords were fully viewed then it was graded as I. If the vocal cords were partially viewed then it was graded as IIA. If only arytenoids and epiglottis seen then it was graded as IIB. If the part of the glottis could not be visualized but the epiglottis could be visualized then it was graded as III. If neither glottis nor epiglottis could be visualized then it was graded as IV [14].

### Intubation difficulty scale score

Intubation Difficulty Scale score was defined as 0–2: easy intubation, 3–4: slight difficult intubation, and 5 or more as moderate or major difficulties in intubation [15].

### MACOCHA score

It was calculated from seven variants (Mallampati score III or IV, apnea syndrome, cervical spine limitation, opening mouth < 3 cm, coma, hypoxia, and non-trained anesthesiologists). Scores are ranged from 0 to 12, with higher the scores a higher the risk of difficult intubation [16].

### Hypoxemia

A decrease in pulse oximetry reading below 80% for at least 5 s during intubation procedure was considered as severe hypoxia [2]. The lowest value of pulse oximetry value during the intubation procedure, the value of pulse oximetry reading at the beginning of preoxygenation, and the reading of pulse oximetry value at the end of preoxygenation were collected.

### Laryngoscopy

Attempts to insert the endotracheal tube (F. Hoffmann-La Roche AG, Basel, Switzerland) into the trachea lasting 10 min or more time using conventional laryngoscopy, duration of laryngoscopy, and the number of laryngoscopy attempt(s) were recorded.

### Complications

Data regarding use of alternative management devices, agitation, immediate complications (arterial hypotension, bradycardia, sustained arrhythmia, esophageal intubation, regurgitation, gastric distension, injuries in the oral cavity, new infiltrate on chest radiograph, cardiac arrest, and death), and late complications (worsening of SOFA (Sepsis-related Organ Failure Assessment; score from days 1 to 7. Scores of SOFA are ranged from 0 to 24, with higher the scores a more severe organ failure [17]), the occurrence of ventilator-associated pneumonia, duration of mechanical ventilation, and length of stay in the intensive care unit) were collected from medical records of institute.

The preoxygenation, intubation, and laryngoscopy were performed by emergency physicians of institute. Also, Outcomes were measured by emergency physicians of institute.

### Statistical analysis

The study was performed with the hypothesis that severe hypoxia could have occurred in 25% of patients during the preoxygenation procedure [11, 12]. The study was enrolled in 315 patients with 80% power (*β* = 0.2) and 5% two-sided type-I error (*α* = 0.05) at a 95% level of confidence and the sample size (minimum patients required in each cohort) was reported 151. SPSS v25.0, IBM Corporation, Armonk, NY, USA was used for statistical analysis purposes. Constant variables are reported as frequency (percentages) and continuous and ordinal variables are demonstrated as mean ± standard deviation (SD). The Fischer exact test was performed for constant variables and the Mann-Whitney *U*-test [2] was performed for continuous and ordinal variables at a two sided *α*-level of 0.05. All outcomes were considered exploratory. All results were considered significant at a 95% confidence level.

## Results

### Study population

**Fig. 1:**
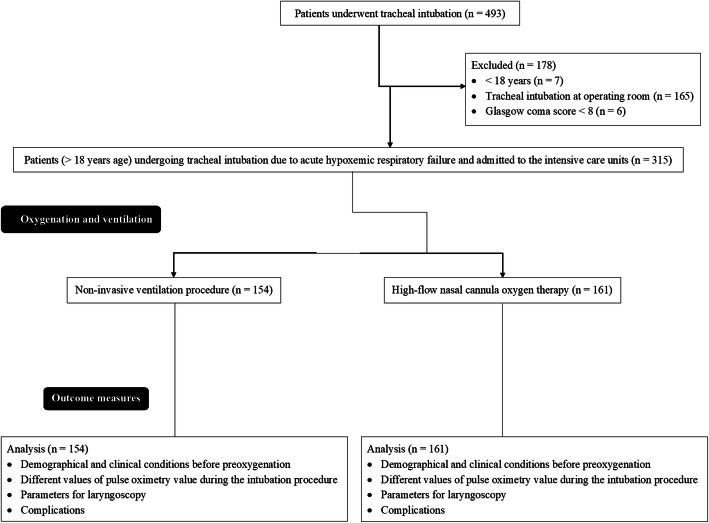
Flow diagram of the oxygenation and ventilation

From 15 January 2018 to 1 October 2019, a total of 493 patients underwent tracheal intubation at the Chinese PLA General Hospital, Beijing, China and the referring hospitals. Among them, seven patients were below 18 years of age, 165 patients underwent tracheal intubation at the operating room, six patients had a Glasgow coma score < 8. Therefore, data of these patients (*n* = 178) were excluded from the analysis. Data of the demographical and clinical conditions before preoxygenation, preoxygenation, intubation procedure, laryngoscopy procedure, and complications during intubation procedure of 315 patients (> 18 years age) undergoing tracheal intubation due to acute hypoxemic respiratory failure admitted to the intensive care units were included in the analysis (Fig. 1). A total of 161 patients had recent laryngeal, esophageal, or/ and substantial facial fractures. Therefore, these patients underwent HCO therapy (HCO cohort) and 154 patients underwent NIV procedure (NIV cohort) for oxygenation and ventilation.

### Demographical and clinical conditions

All enrolled patients had a partial pressure of arterial oxygen to fraction of inspired oxygen ratio less than 300 mmHg and respiratory rates more than 25 breaths/ min. There were no significant differences in the demographical and clinical conditions of the patients before preoxygenation between both cohorts (*p* > 0.05 for all parameters, Table 1). All patients underwent preoxygenation strategies in intensive care units.

**Table 1:** Demographical and clinical conditions of the patients before preoxygenation

Parameters	Cohorts		Comparisons between cohorts
	NIV	HCO	
Procedure for oxygenation and ventilation	Non-invasive ventilation	High-flow nasal cannula oxygen	
Patients included in the analysis	154	161	*p*-value
Age (years)			
Minimum	28	27	0.377
Maximum	68	67	
Mean ± SD	57.12 ± 11.14	58.22 ± 10.91	
Sex			
Male	105 (68)	107 (66)	0.811
Female	49 (32)	54 (34)	
Body-mass index (kg/m^2^)	25.52 ± 1.11	25.22 ± 1.85	0.084
Simplified Acute Physiology Score II	53 ± 13	50 ± 15	0.059
Sepsis-related Organ Failure Assessment score	8.12 ± 4.35	9.01 ± 4.61	0.079
Underlying chronic lung disease	42 (27)	51 (32)	0.459
Upper airway tract cancer history	6 (4)	4 (2)	0.531
Reason for the intensive care unit admission			
Respiratory infection	54 (35)	61 (38)	0.392
Chronic obstructive pulmonary disease exacerbation	10 (7)	11 (7)	
Extra-pulmonary acute respiratory distress syndrome.	15 (10)	12 (7)	
Pulmonary atelectasis	5 (3)	3 (2)	
Shock	42 (27)	57 (35)	
Cardiogenic pulmonary edema	15 (10)	8 (5)	
Neurologic conditions	13 (8)	9 (6)	
Vasopressor support at inclusion	28 (18)	31 (19)	0.885
Bilateral pulmonary infiltrates	77 (50)	82 (51)	0.911
Respiratory rates (breaths/ min)	61 ± 9	62 ± 8	0.298
The partial pressure of arterial oxygen to fraction of inspired oxygen ratio			
Mild hypoxemia (201–300 mmHg)	34 (22)	35 (22)	0.889
Moderate-to-severe hypoxemia (≤200 mmHg)	120 (78)	126 (78)	

Constant variables are reported as frequency (percentages) and continuous and ordinal variables are reported as mean ± standard deviation (SD)

For constant variables the Fischer exact test and for continuous and ordinal variables the Mann-Whitney *U*-test was performed for statistical analysis

A *p*-value of less than 0.05 was considered significant

Fraction of inspired oxygen = 0.21 + oxygen flow rate × 0.03

### Hypoxia

**Fig. 2:**
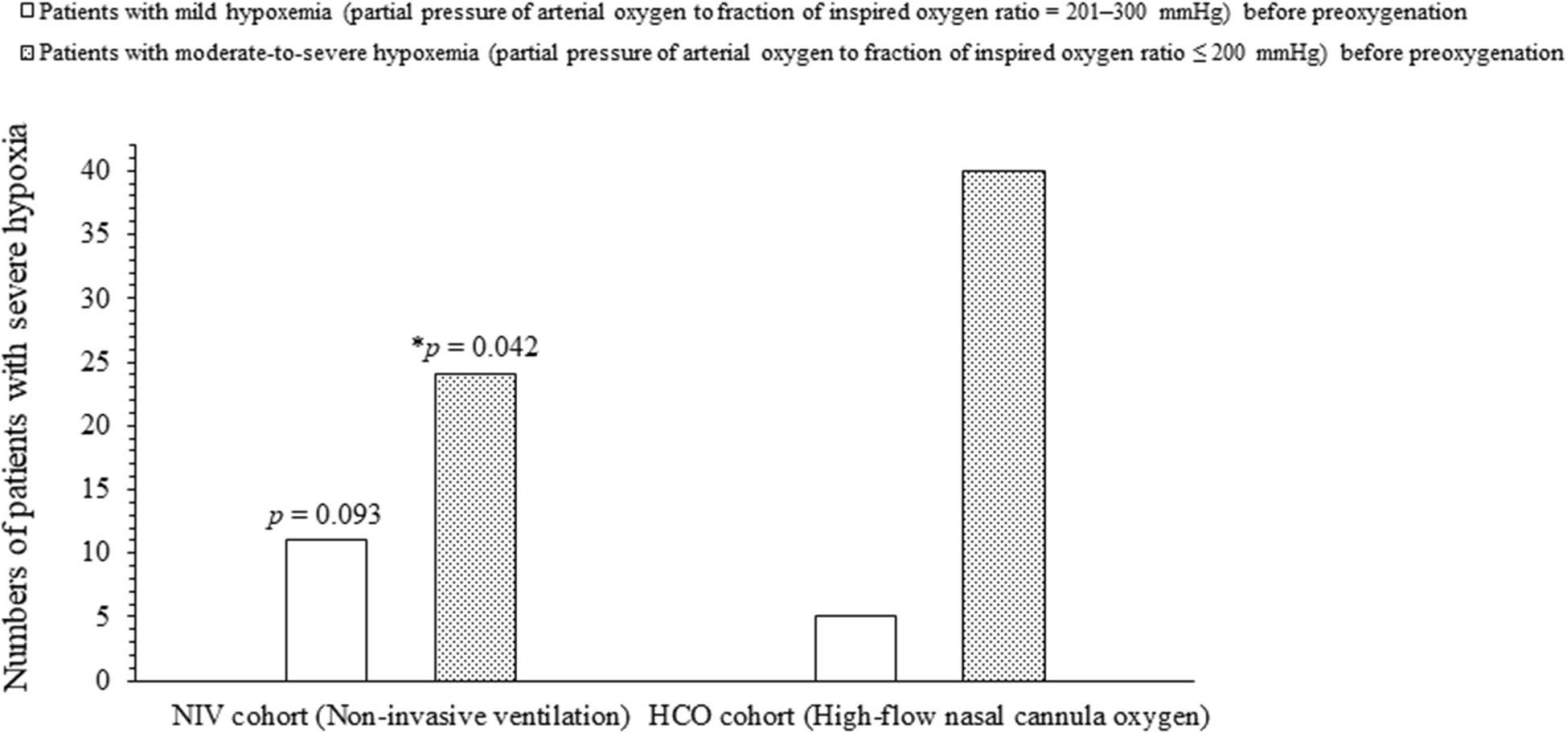
The occurrence of severe hypoxia. Variables are reported as frequency. The Fischer exact test was performed for statistical analysis. A *p*-value of less than 0.05 was considered significant. ^*^Significantly lower than the HCO cohort. Severe hypoxia: A pulse oximetry value < 80% for at least 5 s during intubation procedure. Fraction of inspired oxygen = 0.21 + oxygen flow rate × 0.03

A total of 35 (23%) patients of the NIV cohort had severe hypoxia and 44 (28%) patients of the HCO cohort had severe hypoxia (*p* = 0.303, Table 2) during the intubation procedure. The values of pulse oximetry were reported higher for patients of NIV cohort than those of HCO cohort (Table 3). Severe hypoxia had occurred among 11 (32%) patients of the NIV cohort and 5 (14%) patients of the HCO cohort (*p* = 0.093) who have mild hypoxemia (partial pressure of arterial oxygen to fraction of inspired oxygen ratio = 201–300 mmHg) before preoxygenation. Severe hypoxia had occurred among 24 (20%) patients of the NIV cohort and 40 (32%) patients of the HCO cohort (*p* = 0.042) who have moderate-to-severe hypoxemia (partial pressure of arterial oxygen to fraction of inspired oxygen ratio ≤ 200 mmHg) before preoxygenation (Fig. 2).

**Table 2:** The different values of pulse oximetry value during the intubation procedure

Values of pulse oximetry	Cohorts		Comparisons between cohorts
	NIV	HCO	
Procedure for oxygenation and ventilation	Non-invasive ventilation	High-flow nasal cannula oxygen	
Patients included in the analysis	154	161	*p*-value
Hypoxemia			
At the beginning of preoxygenation (mean ± standard deviation)	95 ± 4%	94 ± 5%	0.052
The lowest value during the intubation procedure (mean ± standard deviation)	78 ± 4%	77 ± 6%	0.084
Numbers of patients with severe hypoxia^a^ (frequency (percentages))	35 (23)	45 (28)	0.303
At the end of preoxygenation (mean ± standard deviation)	97 ± 5%	96 ± 5%	0.077

For constant variables the Fischer exact test and for continuous and ordinal variables the Mann-Whitney *U*-test was performed for statistical analysis

^a^A decrease in pulse oximetry reading below 80% for at least 5 s during intubation procedure

A *p*-value of less than 0.05 was considered significant

**Table 3:** The different values of pulse oximetry value of patients according to different hypoxemia conditions during the intubation procedure

Values of pulse oximetry	Mild hypoxemia (201–300 mmHg)^b^		^a^*p*-value	Moderate-to-severe hypoxemia (≤200 mmHg)^b^		^a^*p*-value
	NIV cohort	HCO cohort		NIV cohort	HCO cohort	
Procedure for oxygenation and ventilation	Non-invasive ventilation	High-flow nasal cannula oxygen		Non-invasive ventilation	High-flow nasal cannula oxygen	
Patients included in the analysis	34	35		120	126	
At the beginning of preoxygenation	96 ± 2%	95 ± 3%	0.109	94 ± 5%	93 ± 5%	0.118
The lowest value during the intubation procedure	79 ± 3%	78 ± 2%	0.107	77 ± 15%	76 ± 12%	0.084
At the end of preoxygenation	98 ± 5%	95 ± 8%	0.067	96 ± 8%	94 ± 8%	0.052

Variables are reported as mean ± standard deviation (SD)

The Mann-Whitney *U*-test was performed for statistical analysis

^a^Comparisons between cohorts

^b^Partial pressure of arterial oxygen to fraction of inspired oxygen ratio

A *p*-value of less than 0.05 was considered significant

### Laryngoscopy

There were no significant differences for the time duration of laryngoscopy (*p* = 0.847) and the number of laryngoscopies attempts (*p* = 0.804) between both cohorts (Table 4).

**Table 4:** The parameters for laryngoscopy

Parameters	Cohorts		Comparisons between cohorts
	NIV	HCO	
Procedure for oxygenation and ventilation	Non-invasive ventilation	High-flow nasal cannula oxygen	
Patients included in the analysis	154	161	*p*-value
Time duration of laryngoscopy (min)			
< 1	87 (56)	96 (59)	0.847
1–3	52 (34)	51 (32)	
> 3	15 (10)	14 (9)	
Number of laryngoscopies attempts			
1	123 (80)	126 (78)	0.804
2	23 (15)	28 (18)	
≥ 3 or procedural time ≥ 10 min	8 (5)	7 (4)	

Variables are reported as frequency (percentages)

The Fischer exact test was performed for statistical analysis

A *p*-value of less than 0.05 was considered significant

### Intubation scoring items

There were no significant differences for MACOCHA score, Modified Cormack-Lehane grade, and Intubation Difficulty Scale score between both cohorts (Table 5).

**Table 5:** Intubation scoring items

Scoring items	Cohorts		Comparisons between cohorts
	NIV	HCO	
Procedure for oxygenation and ventilation	Non-invasive ventilation	High-flow nasal cannula oxygen	
Patients included in the analysis	154	161	*p*-value
MACOCHA score			
< 3	123 (80)	134 (83)	0.469
≥ 3	31 (20)	27 (17)	
Modified Cormack-Lehane grade			
I, IIA, or IIB	139 (90)	144 (89)	0.854
III or IV	15 (10)	17 (11)	
Intubation Difficulty Scale score			
0–2	15 (10)	14 (9)	0.733
3–4	87 (57)	98 (61)	
≥ 5	52 (34)	49 (30)	

Variables are reported as frequency (percentages)

The Mann-Whitney *U*-test was performed for statistical analysis

A *p*-value of less than 0.05 was considered significant

### Immediate and late complications

The most common complications were hypertension, pulmonary aspiration, and increased 30-day mortality. There was no significant difference for immediate complications (Table 6) and late complications (Table 7) between both cohorts during intubation procedure (*p* > 0.05 for all).

**Table 6:** The immediate complications during intubation procedure

Complications	Cohorts		Comparisons between cohorts
	NIV	HCO	
Procedure for oxygenation and ventilation	Non-invasive ventilation	High-flow nasal cannula oxygen	
Patients included in the analysis	154	161	*p*-value
Use of alternative management devices	17 (11)	23 (14)	0.403
Agitation	2 (1)	1 (1)	0.616
At least one episode of systolic arterial hypotension < 90 mmHg	71 (46)	83 (52)	0.368
Bradycardia	3 (2)	5 (3)	0.724
Sustained arrhythmia	4 (3)	3 (2)	0.718
Esophageal intubation	9 (6)	8 (5)	0.806
Regurgitation	1 (1)	2 (1)	0.998
Gastric distension	12 (8)	11 (7)	0.829
Injuries in the oral cavity	1 (1)	2 (1)	0.998
New infiltrate on chest radiograph	29 (19)	33 (20)	0.777
Cardiac arrest	2 (1)	8 (5)	0.105

Variables are reported as frequency (percentages)

The Fischer exact test was performed for statistical analysis

A *p*-value of less than 0.05 was considered significant

**Table 7:** The late occurring complications during intubation procedure

Complications	Cohorts		Comparisons between cohorts
	NIV	HCO	
Procedure for oxygenation and ventilation	Non-invasive ventilation	High-flow nasal cannula oxygen	
Patients included in the analysis	154	161	*p*-value
Sepsis-related Organ Failure Assessment score at 1	9.89 ± 2.52	10.45 ± 3.52	0.107
Sepsis-related Organ Failure Assessment score at 7	6.12 ± 1.22	6.62 ± 3.15	0.066
Ventilator-associated pneumonia	38 (25)	32 (20)	0.344
Duration of mechanical ventilation (days)	11.12 ± 3.45	11.78 ± 3.11	0.075
Length of stay in the intensive care unit (days)	14.11 ± 3.16	14.22 ± 4.12	0.791
Death within a month	43 (28)	51 (32)	0.538

Constant variables are reported as frequency (percentages) and continuous and ordinal data are demonstrated as mean ± standard deviation (SD)

The Fischer exact test was performed for constant data and the Mann-Whitney *U*-test was performed for continuous and ordinal data

A *p*-value of less than 0.05 was considered significant

## Discussion

The study reported that NIV could not change the risk of severe hypoxia and complications during intubation procedure as compared to HCO. The results of the severe hypoxia and complications of the current study were parallel with those of randomized trials [2, 12]. Different oxygenation devices have no different effects on severe hypoxia and other complications during preoxygenation.

The study reported that NIV was reduced the risk of severe hypoxia as compared to HCO among patients with moderate-to-severe hypoxemia [18] before preoxygenation. The results of the risk of severe hypoxia among patients with moderate-to-severe hypoxemia before preoxygenation of the current study were agreed with those of a randomized trial [2]. NIV has beneficial effects on patients with the moderate-to-severe hypoxemic condition before preoxygenation during oxygenation and ventilation.

The current study is reported 25% (80 out of 315) patients with severe hypoxia during preoxygenation. The results of the total numbers of patients reported with severe hypoxia of the current study were consistent with those of randomized trials [2, 10–12] but not consistent with the prospective, controlled study [3]. Accurate analysis of pulse oximetry value is required to record severe hypoxia conditions during preoxygenation. Also, the intubation procedure is performed in an emergency condition. Therefore, it is difficult to detect severe hypoxia conditions during preoxygenation.

At the end of preoxygenation, overall as well as individually for patients with mild hypoxemia and patients with moderate-to-severe hypoxemia [18] the values of pulse oximetry were reported higher for who underwent NIV than those underwent HCO. The results of the pulse oximetry value of the current study consistent with those of randomized trials [2, 9]. NIV improves oxygenation similar to invasive ventilation [2]. While, HCO has a positive end-expiratory pressure effect to improve oxygenation [19], which has a lower intensity than NIV [5, 6]. Also, HCO could be generated 1–3 cm H_2_O a positive end-expiratory pressure to improve oxygenation, that is lower than that generated by NIV [20]. Laryngeal/ esophageal illness/ injuries that led to allocation to HCO. The injuries to these structures confound the results (e.g., the effects that damage to these structures may have action on preoxygenation, laryngoscopy and intubation). The effect of apneic oxygenation during laryngoscopy by NIV is superior to that does by HCO but further research is required to compare NIV with HCO without bias of the inclusion criteria to state the hypothesis clearly.

They study involved a large sample size and can provide important information for management of patients with acute respiratory failure. Still, there are defects in the study, for example, a non-randomized retrospective study, and lack of a control group of preoxygenation with the Bag Valve Mask. The mortality was not the primary outcome of the study but available randomized controlled trials [9–12, 21] have assessed mortality as the primary outcome. Also, mortality is a major outcome in studies for critically ill patients [22]. The treatment effects were not considered on the results. Patients who had a Glasgow coma score < 8 (*n* = 6) were excluded from the analysis. However, data of these patients are unlikely to affect results of the current study. The study was evaluated MACOCHA score in addition to Intubation Difficulty Scale score. The Intubation Difficulty Scale score is used a posteriori and not a priori [23]. Therefore, the study was evaluated MACOCHA score in addition to Intubation Difficulty Scale score. A total of minimum 10 min were chosen to insert the endotracheal tube using conventional laryngoscopy. This arbitrary time frame would not align with best practice nor current evidence. The non-trained physicians were also involved in the study. Therefore, 10 min was required to insert the endotracheal tube into the trachea. Most patients with acute respiratory failure are intubated in emergency department or general ward; it is rare that an acute respiratory failure patient is intubated in the intensive care unit. Before transferring to the intensive care unit, the acute respiratory failure should be corrected, otherwise, there is high risk of cardiac arrest during transportation. The possible justification for the same is that the current study had included patients those were admitted in the intensive care unit and underwent emergency intubation due to the acute respiratory failure.

## Conclusions

When compared, there was no difference between non-invasive ventilation technique and high-flow oxygen therapy to minimize severe hypoxia prior to laryngoscopy and endotracheal intubation in patients with acute respiratory failure. However, the effect of apneic oxygenation during laryngoscopy by non-invasive ventilation is superior to that does by high-flow oxygen therapy especially for patients with moderate-to-severe hypoxemia before preoxygenation.

## Data Availability

The datasets were used and analyzed during the current study available from the corresponding author on reasonable request.

## Abbreviations

HCO: High-flow nasal cannula oxygen therapy (high-flow oxygen)
MACOCHA score: Mallampati score III or IV, apnea syndrome, cervical spine limitation, opening mouth < 3 cm, coma, hypoxia, and non-trained anesthesiologists score
NIV: Non-invasive ventilation
SD: Standard deviation
SOFA: Sepsis-related Organ Failure Assessment
