# Correction to: Comparison of hypoxemia, intubation procedure, and complications for noninvasive ventilation against high-flow nasal cannula oxygen therapy for patients with acute hypoxemic respiratory failure: a nonrandomized retrospective analysis for effectiveness and safety (NIVaHIC-aHRF)

**DOI:** 10.1186/s12873-021-00441-3

**Published:** 2021-04-14

**Authors:** Chao Zhang, Min Ou

**Affiliations:** The Sixth Department of Health Care, The Second Medical Center & National Clinical Research Center for Geriatric Diseases, Chinese PLA General Hospital, Beijing, 100048 China

**Correction to: BMC Emerg Med 21, 6 (2021)**

**https://doi.org/10.1186/s12873-021-00402-w**

The original article [1] incorrectly presented the affiliation for both co-authors. This error has since been corrected.
